# The Effect of Feedback on Resistance Training Performance and Adaptations: A Systematic Review and Meta-analysis

**DOI:** 10.1007/s40279-023-01877-2

**Published:** 2023-07-06

**Authors:** Jonathon Weakley, Nicholas Cowley, Brad J. Schoenfeld, Dale B. Read, Ryan G. Timmins, Amador García-Ramos, Thomas B. McGuckian

**Affiliations:** 1 School of Behavioural and Health Sciences, Australian Catholic University, McAuley at Banyo, Brisbane, Australia; 2 Sports Performance, Recovery, Injury and New Technologies (SPRINT) Research Centre, Australian Catholic University, Brisbane, QLD Australia; 3 Carnegie Applied Rugby Research (CARR) Centre, Carnegie School of Sport, Leeds Beckett University, Leeds, UK; 4 Department of Exercise Science and Recreation, CUNY Lehman College, Bronx, NY USA; 5 Department of Sport and Exercise Sciences, Institute of Sport, Manchester Metropolitan University, Manchester, UK; 6 Department of Physical Education and Sport, Faculty of Sport Sciences, University of Granada, Granada, Spain; 7 Department of Sports Sciences and Physical Conditioning, Faculty of Education, Universidad Católica de la Santísima Concepción, Concepción, Chile; 8 Healthy Brain and Mind Research Centre, School of Behavioural and Health Sciences, Australian Catholic University, Melbourne, Australia

## Abstract

**Background:**

Augmented feedback is often used during resistance training to enhance acute physical performance and has shown promise as a method of improving chronic physical adaptation. However, there are inconsistencies in the scientific literature regarding the magnitude of the acute and chronic responses to feedback and the optimal method with which it is provided.

**Objective:**

This systematic review and meta-analysis aimed to (1) establish the evidence for the effects of feedback on acute resistance training performance and chronic training adaptations; (2) quantify the effects of feedback on acute kinematic outcomes and changes in physical adaptations; and (3) assess the effects of moderating factors on the influence of feedback during resistance training.

**Methods:**

Twenty studies were included in this systematic review and meta-analysis. This review was performed using the Preferred Reporting Items for Systematic Reviews and Meta-Analyses (PRISMA) guidelines. Four databases were searched, and studies were included if they were peer-reviewed investigations, written in English, and involved the provision of feedback during or following dynamic resistance exercise. Furthermore, studies must have evaluated either acute training performance or chronic physical adaptations. Risk of bias was assessed using a modified Downs and Black assessment tool. Multilevel meta-analyses were performed to quantify the effects of feedback on acute and chronic training outcomes.

**Results:**

Feedback enhanced acute kinetic and kinematic outputs, muscular endurance, motivation, competitiveness, and perceived effort, while greater improvements in speed, strength, jump performance, and technical competency were reported when feedback was provided chronically. Furthermore, greater frequencies of feedback (e.g., following every repetition) were found to be most beneficial for enhancing acute performance. Results demonstrated that feedback improves acute barbell velocities by approximately 8.4% (*g* = 0.63, 95% confidence interval [CI] 0.36–0.90). Moderator analysis revealed that both verbal (*g* = 0.47, 95% CI 0.22–0.71) and visual feedback (*g* = 1.11, 95% CI 0.61–1.61) were superior to no feedback, but visual feedback was superior to verbal feedback. For chronic outcomes, jump performance might have been positively influenced (*g* = 0.39, 95% CI − 0.20 to 0.99) and short sprint performance was likely enhanced (*g* = 0.47, 95% CI 0.10–0.84) to a greater extent when feedback is provided throughout a training cycle.

**Conclusions:**

Feedback during resistance training can lead to enhanced acute performance within a training session and greater chronic adaptations. Studies included in our analysis demonstrated a positive influence of feedback, with all outcomes showing superior results than when no feedback is provided. For practitioners, it is recommended that high-frequency, visual feedback is consistently provided to individuals when they complete resistance training, and this may be particularly useful during periods of low motivation or when greater competitiveness is beneficial. Alternatively, researchers must be aware of the ergogenic effects of feedback on acute and chronic responses and ensure that feedback is standardised when investigating resistance training.

**Supplementary Information:**

The online version contains supplementary material available at 10.1007/s40279-023-01877-2.

## Key Points

**Table:** 

When feedback is provided during resistance training, kinetic and kinematic outputs are enhanced, with barbell velocity significantly increasing by approximately 8.4% (*g* = 0.63, 95% confidence interval [CI] 0.36–0.90). Furthermore, improvements in motivation, competitiveness, muscular endurance, and perceptions of effort have been reported to occur.
When feedback was supplied chronically across a training cycle, all studies demonstrated greater improvements in physical qualities (e.g., maximum strength) compared with when feedback was not provided. Furthermore, the meta-analytical outcomes indicated that jump (*g* = 0.39, 95% CI − 0.20 to 0.99) and short sprint performance (*g* = 0.47, 95% CI 0.10–0.84) may have small but meaningful greater improvements when feedback is consistently provided.
Feedback during resistance training is most effective when it is supplied with a high frequency (e.g., following each repetition). The moderator analysis showed no statistically significant difference in the effects of feedback when high or low loads were used, lower or upper body exercises were implemented, mean or peak velocity was supplied, or single or multiple sets were completed. Furthermore, while verbal and visual feedback were superior to no feedback, visual feedback had a statistically greater effect on acute performance than verbal feedback.

## Introduction

Resistance training plays an important role in health and physical performance [1–3]. It can increase lean body mass, strength, and power [4–6], while also decreasing the risk of numerous diseases [2]. When prescribing resistance training, acute programming variables such as intensity, volume, and rest time are often carefully considered as they can influence acute performance and subsequent physical adaptations [7]. For example, the external load that is used during resistance training can alter the kinetic and kinematic outputs (e.g., velocity, power) of an exercise [6, 8, 9] and, if an individual is consistently exposed to heavier or lighter loads, can alter the adaptative response (e.g., strength) [10]. However, an additional consideration that may substantially influence the kinetic and kinematic outputs and subsequent adaptations that occur is the type and amount of feedback that is provided to an individual during resistance training. While there are various types and forms of feedback (with interested readers directed to the review by Salmoni et al. [11]), the focus of this review is augmented feedback (referred to as ‘feedback’ henceforth), which can be defined as feedback from an external source which provides information regarding the result of performance of a task [12].

The provision of feedback during resistance training can have several acute benefits. These include increased barbell kinetic and kinematic outputs [13, 14], improvements in muscular endurance [15], and changes in perceptions of motivation and competitiveness [16]. Furthermore, feedback can reduce the perception of effort that an individual reports during exercise [16]. However, there remains uncertainty regarding whether feedback is most beneficial with light (e.g., < 50% of one repetition maximum [1RM]) or heavy loads (e.g., ≥ 50% 1RM), which measure has the greatest influence (e.g., mean vs peak velocity), the optimal frequency of feedback, and whether different exercises (e.g., ballistic vs non-ballistic; upper vs lower body) benefit more from its use. For example, Pérez-Castilla et al. [17] demonstrated that feedback of concentric barbell velocity following every repetition may be more beneficial for the production of concentric barbell velocity and power than when it is provided at the end of a set. Alternatively, Jiménez-Alonso et al. [18] suggested that the verbal provision of barbell velocity feedback after each repetition may have greater impact during strength-oriented resistance training compared with ballistic resistance training due to changes in motivation, competitiveness, and a shift in the focus of attention from an internal to an external source of information. Consequently, while feedback appears to enhance the quality of training through an increase in barbell velocity, there is still uncertainty surrounding its implementation and the optimal methods of delivery.

As the provision of feedback appears to have beneficial effects on acute performance, several studies have investigated its effects on changes in physical qualities when it is provided throughout a resistance training programme [19–23]. These studies have demonstrated a broad range of positive adaptations, with data indicating that changes in strength, speed, and power are greater when feedback is chronically supplied across a training programme. For example, Weakley et al. [22] demonstrated greater changes in sprint performance in semi-professional rugby union players compared with a training control group when feedback was provided across each repetition of all exercises (Cohen’s *d* effect size [ES]: 0.40 ± 0.21). Additionally, Nagata et al. [19] highlighted the benefits of frequent feedback throughout a training mesocycle on the development and retention of loaded jump performance in university-level rugby union athletes. Despite promising results, the chronic effects of feedback on training outcomes are still poorly understood, as studies that have investigated its effects on changes in physical qualities often suffer from small sample sizes and limited outcome measures. Consequently, to assess the effectiveness of feedback during resistance training, meta-analysing outcomes across training studies may help establish whether consistent use of feedback can provide a tangible benefit beyond training that does not have feedback. This is particularly important as no review has collated and quantified the acute and chronic effects of feedback in resistance training. Thus, the aim of this systematic review and meta-analysis is threefold: (1) to establish evidence for the effects of feedback on acute resistance training performance and chronic training adaptations; (2) to quantify the effects of feedback on acute kinematic outcomes and changes in physical adaptations; and (3) to assess the effects of a range of moderating factors (e.g., load, body region) on the influence of feedback during resistance training.

## Methods

### Search Strategy

Consistent with the Preferred Reporting Items for Systematic Reviews and Meta-Analyses (PRISMA) guidelines for systematic reviews [24], the academic databases SPORTDiscus, CINAHL, Scopus, and MEDLINE were systematically searched in August 2022 to identify English-language peer-reviewed original research studies that investigated the effects of feedback during resistance training on acute performance outcomes and chronic adaptations. Due to differences in database design, studies were identified by searching ‘abstracts, titles, and key words’ in Scopus; ‘All Text’ in SPORTDiscus and CINAHL, and ‘All Fields’ in MEDLINE. The search strategies for each database can be found in Electronic Supplementary Material (ESM) File S1. Medical Subject Headings (MeSH) were not used when searching the MEDLINE database and all search results were extracted and imported into a reference manager (Covidence, Veritas Health Innovation, Melbourne, Australia). A systematic review protocol that includes the review question, search strategy, exclusion criteria, and risk of bias assessment was registered on August 24, 2022, with the Open Science Framework (osf.io/9hnrx).

### Selection Criteria

All duplicate studies were removed automatically by Covidence, and the titles and abstracts of all remaining studies were independently screened for relevance by two researchers (J.W. and N.C). Studies that clearly did not meet the inclusion criteria were removed. Disagreements were resolved through discussion or via an additional researcher (T.M.). The full texts of the remaining studies were then assessed for eligibility. To be eligible for inclusion, studies were required to (1) be original research investigations; (2) be full-text articles written in English; (3) be published in a peer-reviewed academic journal; (4) be an investigation into healthy humans; (5) involve a form of augmented feedback (e.g., visual or verbal) of performance during or following dynamic resistance exercise; (6) report changes in acute performance responses (e.g., velocity, power, total tonnage [kg]) or physical adaptations (e.g., change in strength, sprint performance); and (7) involve an external load that is > 1 kg due to the specific focus on resistance training. If it was deemed that a study did not meet the inclusion criteria, it was excluded from the analysis. The reference lists of all full-text screened studies were manually searched for any studies that were not retrieved in the initial search (i.e., ‘backwards searching’). Additionally, any articles that cited the full-text screened studies were searched (i.e., ‘forwards searching’). If any studies were identified as possibly being eligible for inclusion, they were subjected to the same assessment as previously described.

### Data Extraction and Coding of Outcomes

After determining which studies met the inclusion criteria, two researchers (J.W. and N.C.) separately coded the following variables for each study: authors, title and year of publication, sample size, sex, feedback type, feedback frequency, exercises used, loads used and method of quantification (e.g., kilograms, percentage of one repetition maximum [1RM]), number of sets/reps, kinetic and kinematic outputs, description of the training intervention (duration, intensity, frequency, modality, and type of feedback used), test of physical performance, and mean and standard deviation of physical performance test pre- and post-study. In cases where data were not reported numerically, data were extracted from graphs via WebPlotDigitizer, or the study’s authors were contacted. Coding was cross-checked between reviewers, with any discrepancies resolved by mutual consensus. Consistent with the guidelines of Cooper et al. [25] and used within previous sport science literature [26], 30% of the included studies were randomly selected for re-coding to assess for potential coder drift. Agreement was calculated by dividing the number of variables coded the same by the researchers by the total number of variables. Acceptance required a mean agreement of 0.90 to avoid re-extraction entirely, and after this was met, only those with differing codes were checked and updated. Extracted data were also double-checked by a third researcher (T.M.) prior to analysis.

### Assessment of Reporting Quality

The reporting quality of the research was assessed using a modified version of the Downs and Black checklist [27]. This method is valid for assessing the methodological reporting quality of intervention study designs and has been used extensively in systematic reviews pertaining to sport science [28–30]. Not all assessment criteria were applicable to the studies used in this review; thus, 17 of the 27 criteria were used. These questions can be found in ESM File S2. Study reporting quality was assessed against 17 items, scored as either ‘0’ (unable to determine, or no) or ‘1’ (yes). In total, a score of 17 was indicative of the highest study reporting quality. Values were interpreted on a continuum, with higher scores indicating greater reporting quality.

### Quantitative Synthesis

Analyses were performed using *R* version 4.0.5 and *RStudio* Version 2022.07.2 + 576 [31], effect sizes were calculated using the *esc* package [32], and meta-analyses were performed using the *metafor* package [33]. Pooled Hedges’ *g* effect sizes were interpreted according to conventions of Cohen [34]: 0.2 (small), 0.5 (moderate), 0.8 (large), > 1.0 (very large). Pooled effects were reported with 95% confidence intervals and 95% prediction intervals. Statistical significance was indicated by 95% confidence intervals that did not cross zero.

To quantify the acute effects of feedback on performance, changes in mean and peak velocity output were assessed. This was due to the well-established relationship between load and velocity and the common practice of monitoring velocity to quantify changes in physical capacity [35–38]. It should be noted that other acute outcome measures (e.g., volume load [kg]) which could not be meta-analysed are included within the systematic review portion of this manuscript. For the quantitative assessment of the acute effects of feedback, sample size and mean (*M*) and standard deviation (SD) outcomes for the feedback and control groups were used to calculate Hedges’ *g* effect sizes. Effect sizes were calculated such that positive values would indicate improved performance for the feedback group. Multiple effects were extracted for each study, and therefore effects were not independent. Consequently, multi-level meta-analyses were used to account for the nested data structure [39, 40]. Further, the Hartung–Knapp–Sidik–Jonkman method was used to estimate the variance of pooled effects as it outperforms other methods when there are few studies or substantial heterogeneity [41, 42]. The *I*^2^ statistic was used to assess heterogeneity of effects at the effect size (level 2) and study (level 3) levels. Aggregated effect sizes per study were used to assess publication bias via visual inspection of funnel plots [43]. A series of potential moderators was investigated, including feedback type (verbal vs visual), load (high [i.e., > 50% 1RM] vs low [i.e., ≤ 50% 1RM]), body region (lower body vs upper body), measurement (mean velocity vs peak velocity ([i.e., the two most common forms of acute feedback within the found literature]), and number of sets (multiple vs single sets).

To assess the chronic effects of feedback on physical adaptations, changes in sprint and jump performance were quantified. These physical adaptations were selected due to their relationship with sporting performance and their consistent use throughout the literature which allowed meta-analysis. For the chronic effects of feedback on sprint and jump performance, pre- to post-change in performance was used to compare feedback and control groups. Mean pre- to post-change (*M*_change_) was calculated as post-performance − pre-performance. Pre- to post-standard deviation (SD_change_) was imputed using an accepted formula [44]. Pre- to post-correlation values were not reported by included studies, and a value of *r* = 0.5 was therefore used. Sample size, *M*_change_ and SD_change_ for the feedback and control groups were used to calculate Hedges’ *g* effect size. Effect sizes were calculated such that positive values would indicate improved pre- to post-change in performance for the feedback group.

Separate analyses were conducted for jump performance and sprint performance. For jump performance, multi-level meta-analysis was used to account for the nested data structure. When *I*^2^ was > 50%, sensitivity analysis was performed to investigate potential sources of heterogeneity. Here, an outlier effect was identified (see ESM File S3) and level 3 *I*^2^ = 85.8%. Therefore, the outlier effect was removed to determine if the effect was causing the substantial heterogeneity. Removal of the outlier resulted in acceptable heterogeneity, and therefore the model without the outlier was retained. For sprint performance, multi-level meta-analysis was used to account for the nested data structure. For the included studies, sprint performance was measured at multiple distances within a single sprint (e.g., 10-m, 20-m, and 30-m performance measured using a single 30-m trial [20]). Although multi-level meta-analysis was already being used to account for correlated observations, random-effects meta-analysis using a single effect from each study was also performed (see ESM File S4). Results did not differ substantially from analysis including all effects, and therefore the multi-level meta-analysis was retained.

## Results

### Identification of Studies

**Fig. 1:**
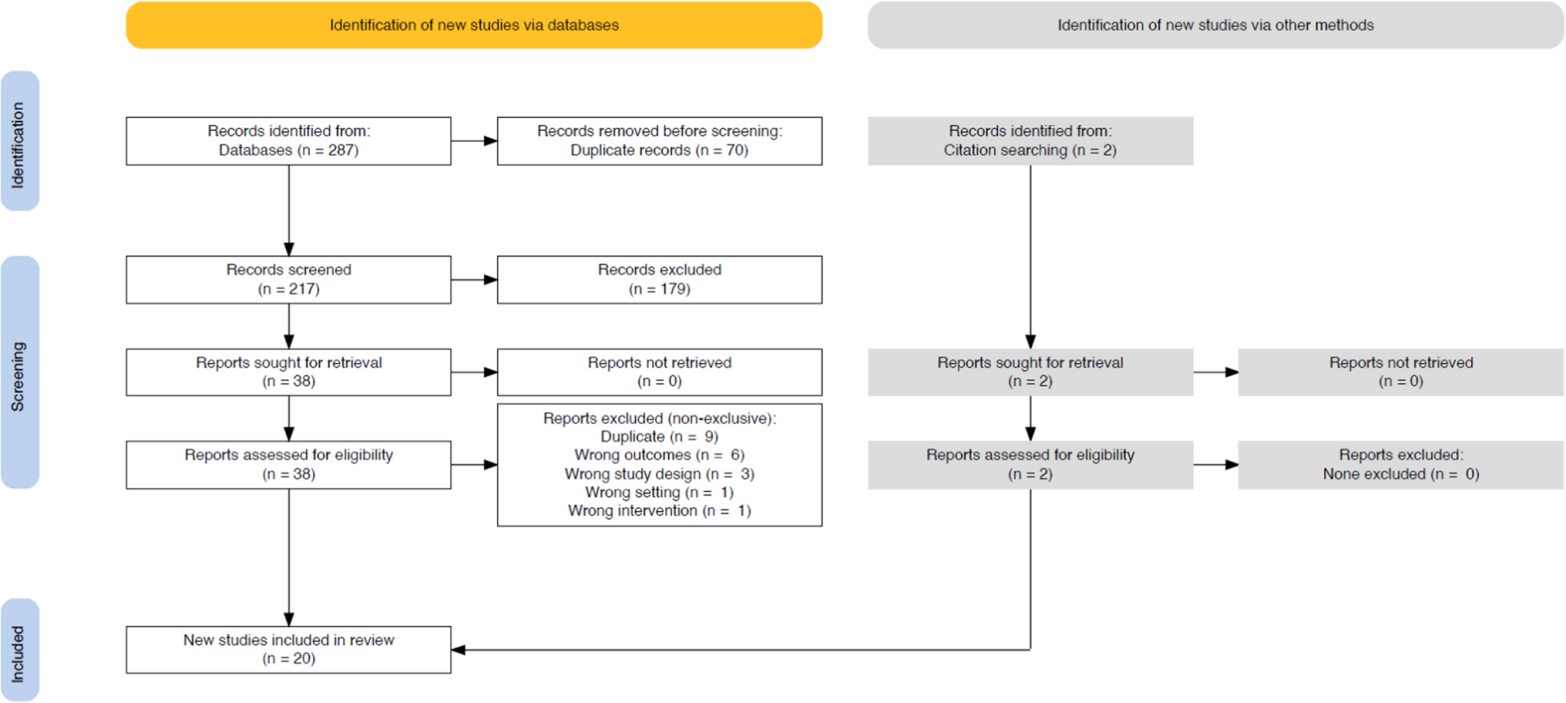
PRISMA flow diagram detailing inclusion and exclusion of manuscripts

The systematic search retrieved a total of 287 studies with zero manuscripts found through screening of reference lists. Seventy of the identified studies were removed as duplicates. The titles and abstracts of the remaining 217 studies were screened, with 38 manuscripts sought for full-text screening. Two additional studies were found through screening of full-text reference lists. During full text review, 20 studies were deemed to meet the inclusion criteria with 13 demonstrating the acute effects of feedback on resistance training performance and seven reporting the chronic effects. The search and screening process is outlined in Fig. 1.

### Research Reporting Quality

The methodological reporting quality of the research investigating the effects of feedback on acute resistance training performance and chronic adaptations was (mean ± SD) 14.5 ± 2.3 and 12.33 ± 3.0, respectively (ESM File S5). Items that were consistently not achieved included questions 10 (relating to the calculation of statistical power) and 27 (relating to all appropriate statistical values being reported).

### Study Characteristics

Of the 20 studies involved in this systematic review and meta-analysis, 13 investigated the acute effects of feedback while seven investigated the chronic effects of feedback (refer to Tables 1 and 2, respectively). Furthermore, 15 used only male participants [13, 14, 16–23, 45–49], two used only female participants [50, 51], two used both males and females [52, 53], and one study did not specify participant sex [15].

**Table 1:** Summary of acute feedback studies included in the systematic review

Study	Participants	Feedback type(s)	Exercise(s) and load(s)	Outcomes
Argus et al. 2011 [13]	9 males Age: 22.1 ± 2.1	Verbal mean velocity feedback following each repetition	Bench throw at 40 kg	Peak power and peak velocity
Campenella et al. 2000 [52]	15 males and 15 females Age: 25.4 ± 2.4	Visual torque graph display after each repetition with verbal encouragement Visual torque graph display after each repetition	Leg extension at 60 deg·s^−1^ Leg flexion at 60 deg·s^−1^	Peak leg extension torque and peak leg flexion torque
Chalker et al. 2018 [47]	*Feedback group 1* 24 males Age: 18.3 ± 3.5 *Feedback group 2* 20 males Age: 18.9 ± 4.6	Visual time-force output display during each repetition	Bodyweight Nordic hamstring exercise	Peak force and inter-limb asymmetry
Ekblom and Eriksson 2012 [50]	7 females Age: 21.4 ± 1.1	Visual EMG feedback	Leg extension at 20 deg·s^−1^ Leg flexion at 20 deg·s^−1^	Mean torque and EMGrms
Hopper et al. 2003 [51]	*Feedback group 1* 8 females Age: 18.6 ± 1.66 *Feedback group 2* 8 females Age: 21.1 ± 1.7	Visual power output feedback after each repetition	45 deg. leg press at 50 kg	Power output
Jiménez-Alonso et al. 2022 [18]	15 males Age: 20.5 ± 3	Verbal mean velocity feedback following each repetition	Bench press at 40%, 55% and 70% 1RM	Mean velocity
Jiménez-Alonso et al. 2022 [46]	17 males Age: 20.2 ± 2.7	Verbal mean velocity feedback following each repetition	Bench press at 75% 1RM Back squat at 75% 1RM Bench throw at 30% 1RM Jump squat at 30% 1RM	Mean velocity and peak velocity
Kimura et al. 1999 [53]	15 males and 15 females Age: 27.3	Visual torque graph display after each repetition with verbal encouragement Visual torque graph display after each repetition	Leg extension at 60 deg·s^−1^ Leg flexion at 60 deg·s^−1^	Peak leg extension torque and peak leg flexion torque
Ok and Bae 2019 [15]	8 (gender N/A) Age: 21.0 ± 0.42	Visual mean velocity feedback following each repetition	Back squat at 65% and 85% 1RM	Number of repetitions, total work, volume load, peak power, peak force, and peak velocity
Pérez-Castilla et al. 2020 [17]	15 males Age: 19.9 ± 2.7	Verbal mean velocity feedback following each repetition, half a set or complete set	Bench throw at 30% 1RM Jump squat at 30% 1RM	Peak velocity
Weakley et al. 2020 [14]	12 males Age: 21.8 ± 0.9	Verbal and visual mean velocity feedback following each repetition	Back squat at 69.8% 1RM	Mean velocity and conscientiousness
Weakley et al. 2019 [16]	15 males Age: 17.1 ± 0.5	Visual mean velocity feedback following each repetition	Back squat at 60.5% 1RM	Mean velocity, subjective motivation and competitiveness, and NASA-TLX
Wilson et al. 2017 [45]	15 males Age: 17.1 ± 0.5	Visual mean velocity feedback following each repetition	Back squat at 60.5% 1RM	Mean velocity, subjective motivation and competitiveness, and NASA-TLX

*1RM* one repetition maximum, *deg*^*.*^*s*^*−1*^ degrees per second, *EMG* electromyographic, *EMGrms* electromyographic root-mean-square, *NASA-TLX* National Aeronautics and Space Administration Task Load Index

**Table 2:** Summary of chronic feedback studies included in the systematic review

Study	Participants	Feedback type(s)	Outcome measures	Outcomes
Nagata et al. 2020 [19]	*Immediate feedback group:* 9 Collegiate rugby players *Average feedback group:* 10 Collegiate rugby players *Visual feedback group:* 10 Collegiate rugby players *Control group:* 8 Collegiate rugby players Age: 20.89 ± 0.8	Verbal velocity feedback following each repetition Visual mean velocity feedback following each set Video recording of each repetition	30-kg squat jump velocity	Immediate verbal feedback showed the greatest improvements in squat jump velocity and retainment across a 4-week period
Randell et al. 2011 [20]	*Feedback group:* 7 Professional rugby players Age: 25.7 ± 3.6 *Control group:* 6 Professional rugby players Age: 24.2 ± 2.5	Visual velocity feedback following each repetition	Vertical jump, broad jump, 10-m, 20-m, 30-m sprint	Feedback group tended to show superior improvements in physical qualities across a 6-week period
Sakadjian et al. 2014 [49]	*Action observation group:* 8 State-level Australian Football players	Verbal coaching cues and observation of video demonstration by a skilled model before each set Verbal coaching cues before each set	Power clean peak power output; power clean technique analysis	Action observation group showed superior improvements in power clean peak power output and technique across a 4-week period
Vanderka et al. 2020 [21]	*Feedback group:* Strength-trained males Age: 22.9 ± 2.2 *Control group:* Strength-trained males Age: 23 ± 2	Visual power output feedback after each repetition	30- and 50-m sprint; 20-m flying sprint; 3RM back half squat; loaded squat jump power max (W and load); CMJ; squat jump	Feedback group showed superior improvements in physical qualities across a 6-week training period
Weakley et al. 2019 [22]	*Feedback group:* 16 Semi-professional rugby players Age: 21 ± 1 *Control group:* 12 Semi-professional rugby players Age: 21 ± 2	Visual and verbal velocity or displacement feedback following each repetition	CMJ; broad jump; 3RM back squat and bench press; 10- and 20-m sprint	Feedback group tended to show superior improvements in physical qualities across a 4-week training period
Winchester et al. 2005 [48]	*Feedback group:* 18 NCAA Division III athletes Age: 22.22 ± 2.13	Visual feedback with video recording and verbal coaching cues	Power clean peak power output, peak force, and bar-path kinematic variables	Improvements were seen in kinetic and kinematic outcomes across a 4-week period
Winchester et al. 2009 [23]	*Feedback group:* 12 NCAA Division I football players *Control group:* 12 NCAA Division I football players Age: 21.72 ± 1.94	Visual feedback with video recording and verbal coaching cues	Power snatch peak power output, peak force, and bar-path kinematic variables at 50%, 70% and 90% 1RM	Feedback group showed superior improvements in kinetic and kinematic outcomes across a 4-week period

*1RM* one repetition maximum, *3RM* three repetition maximum, *CMJ* countermovement jump, *W* watts

In the acute studies, the most commonly investigated exercises were squat [13–18, 45] and bench press variants [13, 17, 18, 46]. However, leg extension and flexion in an isokinetic dynamometer [50, 52, 53], Nordic hamstring curl [47], and the leg press [51] were also investigated. While the majority of studies investigated the effects of feedback following every repetition, the frequency of feedback was also considered, with velocity outcomes being provided at the halfway point of a set and as an average of the entire set [17]. Furthermore, the effect of visual and verbal kinetic or kinematic feedback was provided within each study although the effect of visual kinematic feedback was considered with and without additional verbal encouragement in a single study [53]. Finally, while most free weight and machine-based exercises dictated load as a percentage of maximum [14–18, 45, 46], two studies used a predefined load [13, 51] and one used body mass [47].

All studies that investigated the chronic effects of feedback on performance were carried out across a 4- to 6-week training period [19–23, 48, 49]. Furthermore, six of the seven studies investigated adaptations when only providing feedback on a single exercise [19–21, 23, 48, 49], while only a single study investigated training adaptations when feedback was provided following each exercise [22]. Three studies used only the jump squat (i.e., an exercise that utilises the stretch shortening cycle) or squat jump (i.e., an exercise that mitigates the effects of the stretch shortening cycle) exercise [19–21], while three studies used weightlifting derivatives [23, 48, 49]. The countermovement jump [21, 22] and broad jump [20, 22] were the most commonly used jump variants assessed, but the squat jump [21] and vertical jump (i.e., a countermovement jump with an arm swing) [20] were assessed in a single study each. Short sprint performance was quantified between distances of 0–50 m in three studies [20–22], while three repetition maximum (3RM) strength performance in the back squat [21, 22] and bench press [22] were the only maximum strength exercises investigated. Finally, peak force and power outputs across a range of submaximal loads in the power clean and snatch were assessed in three studies [23, 48, 49].

### Meta-analysis

**Fig. 2:**
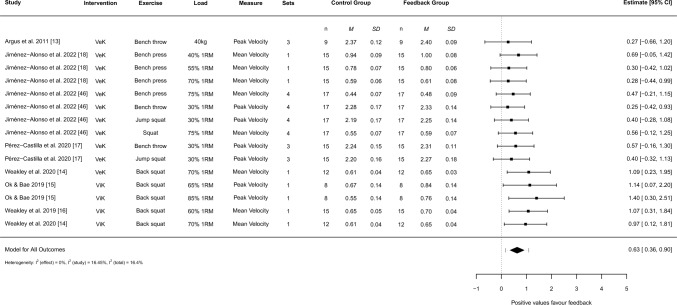
Forest plot demonstrating the acute effects of augmented feedback on velocity outputs during training. *1RM* one repetition maximum, *95% Cl* 95% confidence limit, *kg* kilograms, *M* mean velocity output, *n* participant number, *SD* standard deviation, *VeK* verbal kinematic feedback, *ViK* visual kinematic feedback

For acute performance (Fig. 2), a moderate-strong pooled effect was found, favouring feedback (*g* = 0.63, 95% CI 0.36–0.90, *I*^2^ [total] = 16.4%). Moderator analysis (Table 3) revealed that both verbal (*g* = 0.47, 95% CI 0.22–0.71) and visual feedback (*g* = 1.11, 95% CI 0.61–1.61) were superior to no feedback, but that visual feedback was significantly better than verbal feedback (*p* = 0.027). Moderator analyses for load (*p* = 0.215), body region (*p* = 0.089), measurement (*p* = 0.552), and number of sets (*p* = 0.137) were all statistically non-significant. The funnel plots of aggregated effects did not reveal evidence of publication bias (ESM File S6 and ESM File S7).

**Table 3:** Moderator analysis of feedback variables

Moderator	*k*	*n*	Hedges' g	95% CI	95% PI	*SE*	Weight (%)	*p*-value
Baseline	7	15	0.63	0.36 to 0.90	0.17 to 1.09	0.13	100	
Intervention	7	15						0.027
Verbal	5	11	0.47	0.22 to 0.71	0.22 to 0.71	0.11	76	
Visual	3	4	1.11	0.61 to 1.61	0.61 to 1.61	0.23	24	
Load	7	15						0.215
High	5	9	0.71	0.41 to 1.01	0.38 to 1.04	0.14	57	
Low	4	6	0.44	0.11 to 0.78	0.08 to 0.81	0.16	43	
Body region	7	15						0.089
Lower	5	8	0.78	0.47 to 1.09	0.47 to 1.09	0.15	51	
Upper	4	7	0.41	0.10 to 0.71	0.10 to 0.71	0.14	49	
Measurement	7	15						0.552
Mean velocity	4	8	0.7	0.33 to 1.08	0.11 to 1.30	0.17	56	
Peak velocity	4	7	0.56	0.16 to 0.97	− 0.05 to 1.18	0.19	44	
Sets	7	15						0.137
Multiple	3	7	0.42	0.04 to 0.81	− 0.11 to 0.96	0.18	49	
Single	4	8	0.82	0.44 to 1.20	0.29 to 1.35	0.18	51	

**Fig. 3:**
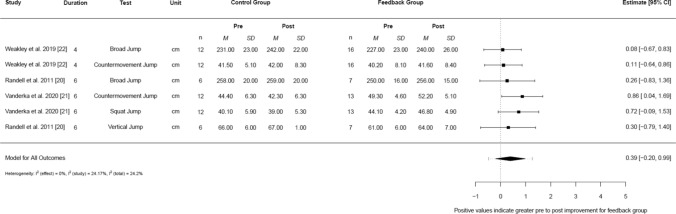
Forest plot demonstrating the chronic effects of augmented feedback on jump performance. *95% Cl* 95% confidence limit, *cm* centimetre, *M* mean velocity output, *n* participant number, *SD* standard deviation

**Fig. 4:**
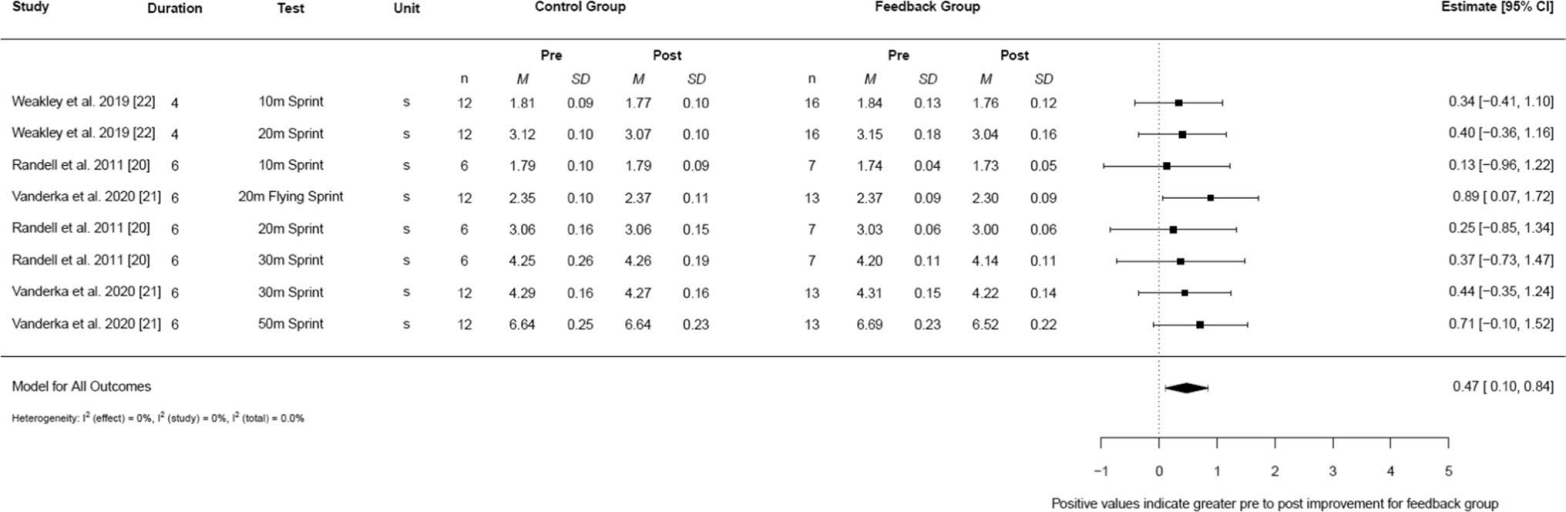
Forest plot demonstrating the chronic effects of augmented feedback on sprint performance. *95% Cl* 95% confidence limit, *m* metre, *M* mean velocity output, *n* participant number, *s* seconds, *SD* standard deviation

For chronic jump performance (Fig. 3), a small-moderate pooled effect was found, but this was not statistically significant (*g* = 0.39, 95% CI − 0.20 to 0.99, 95% PI − 0.48 to 1.26, *I*^2^ [total] = 24.2%). For chronic sprint performance (Fig. 4), a moderate pooled effect was found, favouring feedback (*g* = 0.47, 95% CI 0.10–0.84, 95% PI 0.10–0.84, *I*^2^ [total] = 0%).

## Discussion

The aims of this systematic review and meta-analysis were to (1) establish the evidence for the effects of feedback on acute resistance training performance and chronic training adaptations; (2) quantify the effects of feedback on acute kinematic outcomes and changes in physical adaptations; and (3) assess the effects of a range of moderating factors (e.g., load, body region) on the influence of feedback during resistance training. Of the 13 acute studies that met inclusion criteria, our results demonstrate that regular visual or verbal feedback can enhance training performance with greater force, velocity, power, volume, and repetitions completed. This is supported by the meta-analysis demonstrating that participants are able to express greater velocity outputs (*g* = 0.63, 95% CI 0.36–0.90) when provided feedback across a range of heavy and light loads using upper and lower body exercises.

**Fig. 5:**
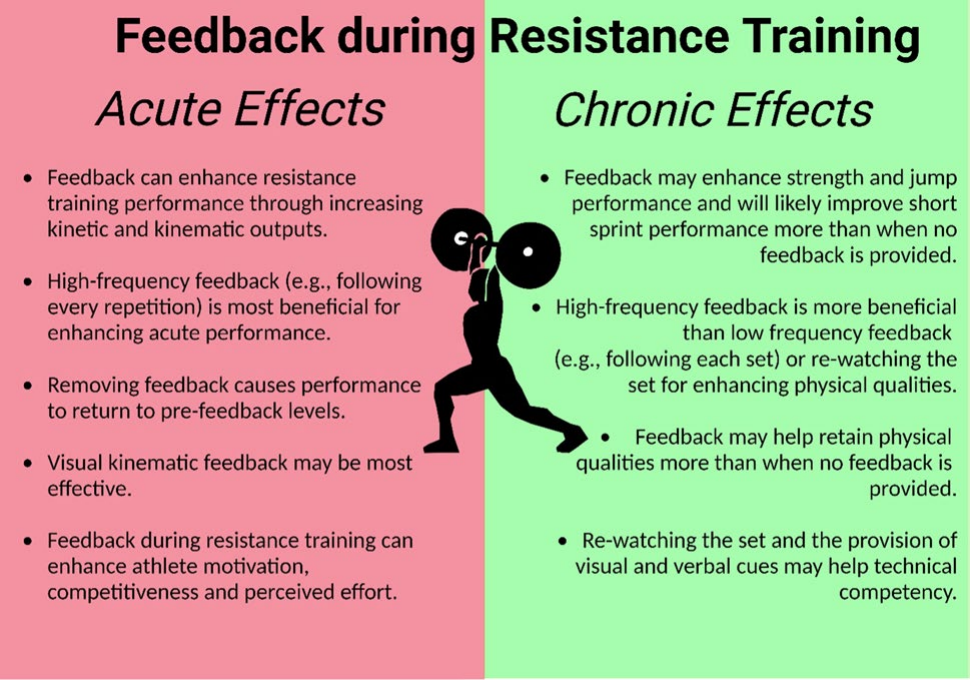
Summary of the acute and chronic effects of feedback during resistance training

The effects of feedback on chronic adaptations tended to support the acute findings, with all studies reporting either greater strength, power, speed, or lifting competency when feedback is provided during training. The meta-analytical outcomes suggested that the provision of feedback can provide meaningful advantages and this can manifest in superior jump and short sprint performance across a training programme. Collectively, these findings demonstrate that the regular provision of feedback is an effective and efficient ergogenic aid that elicits improvements in resistance training performance and can lead to superior adaptations. Considering that feedback can easily be implemented into training and no study shows a detrimental effect, practitioners who wish to maximise athlete training performance and subsequent adaptations are strongly recommended to provide regular, ongoing visual or verbal kinetic or kinematic feedback. Additionally, researchers should be aware of this powerful ergogenic aid and ensure that the provision of feedback during resistance training research is carefully standardised. Figure 5 provides a brief overview of the effects and considerations of feedback during resistance training.

### Acute Training Responses

Of the 13 studies that investigated the effects of feedback on acute resistance training performance, all studies demonstrated a beneficial effect of feedback provision. This is despite the fact that all studies required participants to give ‘maximal’ effort during both feedback and non-feedback conditions. Of note, it appears that feedback is most effective at improving acute resistance training performance when it is provided following each repetition [17]. Furthermore, the addition of verbal encouragement on top of visual or verbal kinematic feedback does not appear to provide any additional benefit [52, 53]. However, it should be noted that when athletes are provided feedback and then it is taken away, performance immediately returns to non-feedback levels [47, 52, 53]. This agrees with previous non-loaded, plyometric research by Keller et al. [54], who showed that augmented feedback can cause immediate improvements in drop jump performance that are lost once feedback is removed. Thus, to maximise resistance kinetic and kinematic outputs, it is recommended that practitioners provide frequent (i.e., following each repetition) and ongoing feedback throughout training.

Several mechanisms have been used to explain why improvements in resistance training performance occur when feedback is provided. Specifically, improvements in motivation and competitiveness have been reported to occur when visual feedback is given [16, 45]. These changes in psychological state have been shown to enhance velocity and power output during both resistance training [16, 45] and non-loaded plyometric [55] exercise. Further, feedback during resistance training has been reported to reduce perceived physical demand [16], and the reported changes in motivation and competitiveness appear to mitigate the acute effects of fatigue across an exercise set [15]. This can enable athletes to complete a greater number of repetitions, and subsequently greater volume, prior to reaching the point of concentric failure [15]. Consequently, it is plausible that the greater kinetic and kinematic outputs that are commonly observed with the provision of feedback [14, 17, 18, 46] are made possible through improved psychological state [16, 45, 55] and reductions in perceptions of physical demand [16, 55].

The meta-analysis of acute outcomes demonstrated that feedback causes an immediate improvement of approximately 8.4% in concentric velocity during resistance training (*g* = 0.63, 95% CI 0.36–0.90). Mean and peak velocity are commonly monitored during resistance training as they are closely related to physical capacity due to their reliable output [9, 38, 56] and linear relationship with load [57–59]. All studies showed a beneficial effect of feedback, despite participants being asked to provide ‘maximal’ effort during each repetition. This shows that feedback is an effective method of enhancing physical performance during resistance training and can cause immediate improvements in kinetic and kinematic outputs. Greater intent and kinematic outputs during training have been linked to enhanced physical adaptation in strength and power outcomes [60, 61] and these findings help to explain the superior chronic adaptations that have been observed throughout the literature [19, 21, 22].

The moderator analysis showed no statistical differences in whether high (i.e., > 50% 1RM) or low (≤ 50% 1RM) loads were used, upper or lower body exercises were completed, mean or peak velocity were supplied, or whether single or multiple sets were employed. However, visual feedback of kinematic data was found to have a statistically greater influence on velocity outputs than verbal feedback (refer to Table 3). Pairing this information with findings from Nagata et al. [19] and Pérez-Castilla et al. [17] that demonstrated the importance of frequency of feedback (i.e., following every repetition) on performance, it is recommended that visual feedback of mean or peak concentric velocity is used consistently across a range of resistance training exercises and loads when aiming to maximise kinetic and kinematic outputs. Furthermore, researchers must be aware that feedback can substantially enhance performance, and this should be carefully standardised when monitoring changes in physical capacity.

### Chronic Training Responses

Seven studies have investigated the effects of feedback on chronic training outcomes, with all interventions occurring across 4- to 6-week mesocycles. Four studies investigated the effects of verbal or visual feedback on changes in sprint, jump, or maximal strength [19–22], while three used a combination of verbal coaching cues and visual feedback to quantify changes in performance of the power clean or power snatch [23, 48, 49]. Similar to the studies that investigated acute outcomes, feedback was largely found to augment adaptations above and beyond what occurs when feedback is not consistently provided during training. Furthermore, no study demonstrated that feedback impaired training adaptations compared with a training control group. It should also be noted that while technology (e.g., linear position transducers, video footage) was commonly used to provide immediate feedback, other simple methods of feedback, such as distance jumped and sprint times, were also provided within training programmes [22]. This suggests that a range of methods can be used within a training mesocycle to provide feedback to athletes and that even small concerted periods of exposure can provide substantial benefit.

Feedback was found to enhance jump performance in all studies that assessed changes across a training programme [19–22]. Furthermore, the meta-analysis found small, but non-significant, increases in in jump performance across a training mesocycle (*g* = 0.39, 95% CI − 0.20 to 0.99) [19–22]. It is feasible that the larger observed improvements in strength [21, 22] may have influenced these improvements in jump results, as the ability to exert force is fundamental to ballistic performance [62]. Additionally, it is likely that the chronic exposure to greater barbell velocities, and subsequently power outputs, during ballistic exercises [19–21] allowed athletes to expose themselves to a greater training stimulus. This reflects the acute findings of the meta-analysis and helps emphasise that improvements in acute training stimuli may lead to enhanced training adaptations. It should be acknowledged that a single study [19] that assessed changes in jump performance was removed from the meta-analysis due to the sensitivity analysis demonstrating the extreme nature of the findings. However, with these findings included (ESM File S3), it was demonstrated that feedback may promote even greater changes in jump performance.

The effects of feedback during resistance training were clearly observed on changes in short sprint performance (i.e., ≤ 50 m), with statistically significant *small to moderate* improvements compared with training in the absence of feedback (*g* = 0.47, 95% CI 0.10–0.84). Furthermore, due to the narrow width of the prediction intervals reported (95% PI 0.10–0.84), practitioners can also be confident that future resistance training interventions that use feedback will induce a significant superior improvement as well. As demonstrated within the current systematic review findings, greater changes in strength and power were consistently reported with the provision of feedback, and it is well established that the ability to rapidly exert force is fundamental to acceleration [63, 64]. Thus, it could be reasonable to speculate that the observed changes in strength and power underpinned these changes in short sprint performance. It should be acknowledged that when outcomes were limited to a single testing distance (i.e., 20-m distance [20–22]) (ESM File S4), near identical findings were reported. Consequently, for practitioners who wish to maximise acceleration and speed in their athletes, it is strongly recommended that feedback is consistently provided during resistance training as this will promote greater short distance sprint adaptations.

### Limitations and Future Directions

While this is the first systematic review and meta-analysis to demonstrate the acute and chronic effects of feedback on resistance training performance and adaptations, several limitations and future directions should be acknowledged. First, due to the relatively small number of studies that have investigated the chronic effects of resistance training with feedback on training adaptations, only jump and short sprint performance outcomes could be assessed. Naturally, practitioners are often interested in additional physical qualities (e.g., strength) but due to the breadth of outcomes reported, it was not possible to ascertain the effect of feedback on these outcomes. It should be noted that despite the inability to meta-analyse certain outcomes, findings from the systematic review can help guide practitioners in whether feedback would enhance adaptations in non-meta-analysed outcomes. For example, 3RM strength in the back squat was assessed by both Weakley et al. [22] and Vanderka et al. [21], with both studies demonstrating that the feedback groups had greater improvements than their corresponding non-feedback groups. Therefore, these findings may still be useful for practitioners. Second, due to the aims of the current study, it was not feasible to investigate effects of feedback on non-loaded plyometric outcomes. However, it is likely that comparable benefits occur, with previous research indicating that there are similar improvements in acute and chronic outcomes [54, 55, 65, 66]. Third, due to the relatively homogenous nature of the participants in the chronic studies, further research that investigates chronic adaptations in young, old, and female participants may be warranted to fully elucidate the effects of feedback. Finally, further studies may continue to investigate the effects of different forms of feedback on acute and chronic outcomes. However, it should be acknowledged that the ‘optimal’ method of feedback may be highly dependent upon the individual. Previous research [14] has indicated that athletes may have a preference as to the form of feedback, and this may be influenced by personality traits (e.g., athletes who demonstrate low levels of conscientiousness may benefit most from encouraging statements from a practitioner).

### Practical Applications

Findings from this systematic review and meta-analysis demonstrate that the provision of feedback during resistance training can be a potent tool for acutely enhancing performance and chronically improving adaptations. Consequently, researchers and practitioners should be aware of its effects and how they can be used to ensure better performance, standardisation, and training outcomes. In the acute setting, feedback may be particularly useful to help drive intent and enhance kinetic and kinematic outputs. In athletes who are technically competent, this can be useful in helping to enhance the stimulus that is applied and may lead to the superior physical adaptations that have been reported throughout the literature. Alternatively, in athletes with limited resistance training experience, some forms of feedback (e.g., model demonstration through video) may support the learning of complex resistance training exercises (e.g., the power clean) and this may be useful for coaches who work with large groups of athletes [11, 49]. Furthermore, the provision of feedback may be useful in helping to improve certain psychological traits that may be beneficial for performance. For example, motivation and competitiveness can be enhanced when feedback is provided. This may not only lead to greater kinetic and kinematic outputs but may also be useful in increasing the total volume that can be completed [15] and reducing the perceived physical demand of the resistance training exercise [16].

When monitoring and testing athletes, however, researchers and practitioners should also be aware of the effects of feedback. Due to the clear effects of feedback on acute performance, common assessments of performance which are used to monitor strength and power adaptations and guide training prescription, such as load-velocity profiles [8, 57, 67] and maximal effort against a set load [15, 35, 68], may be substantially altered. Consequently, when aiming to use kinetic or kinematic data from a resistance training session to infer changes in performance, it is strongly recommended that feedback is standardised, as the improvements in acute performance that are observed when feedback is provided are often larger than the typical between-day changes in performance that are commonly reported [29, 67, 69, 70]. An example of this could be if feedback is provided during testing (e.g., when developing a load-velocity profile) but not training, athletes could be perceived to be substantially underperforming or weaker than they truly are.

The current findings demonstrate that practitioners can confidently implement feedback into resistance training to enhance physical adaptations. The systematic review demonstrated that all physical qualities that were assessed had larger improvements with feedback than when no feedback was provided, and the meta-analysis demonstrated that jump and sprint performance can be enhanced with its use. Furthermore, it is important to recognise that feedback was not found to be detrimental under any conditions and that the improvements reported were above and beyond those that were reported with regular, supervised training prescription in highly trained athletes [19, 20, 22].

In practice, feedback can be provided through a range of different methods, with the greatest benefits seen when it is given with high frequency (i.e., following every repetition) [17, 19] and potentially when kinematic feedback is provided visually. However, athlete preference and feasibility should take precedence when deciding how and when feedback is provided. Despite this, a range of simple methods of giving feedback have been used within resistance training programmes that include the provision of mean/peak velocity, distance jumped, sprint time, and video and coaching cues [22, 23, 48]. Thus, practitioners may wish to selectively implement feedback during exercises that benefit from greater kinetic and kinematic outputs (e.g., plyometric and or exercises that require rapid force expression) or during periods that can benefit from increased motivation and intent.

## Conclusions

This systematic review and meta-analysis demonstrates clear benefits to performance and adaptation when feedback is supplied during resistance training. In all studies within the review, feedback was found to augment performance and adaptation beyond that observed with no feedback and there were no detrimental effects reported. Furthermore, when feedback was provided, there were no statistical differences in performance outcomes when high (i.e., ≥ 50% of 1RM) or low loads were used, upper or lower body exercises were assessed, or when mean or peak velocity was provided across single or multiple sets. However, there may be slight benefits of providing kinematic feedback visually compared with verbally. From the studies included within this review, it was apparent that the frequency of feedback was an important consideration, with greater frequencies being substantially more effective for performance and adaptation compared with lower frequencies (e.g., average set velocity). It was clear that feedback can improve resistance training kinetic and kinematic outputs during training beyond normal maximal intent and these greater outputs may help drive greater performance adaptations. While a range of physical qualities were assessed within the literature (e.g., strength), the meta-analysis demonstrated that changes in jump and short sprint performance tended to be greater when feedback was consistently supplied. It should be noted that these changes are above and beyond regular training responses and demonstrate the potency of feedback to augment training adaptations.

## Supplementary Information

Below is the link to the electronic supplementary material.

Supplementary file1 (DOCX 13 KB)

Supplementary file2 (DOCX 24 KB)

Supplementary file3 (PDF 6 KB)

Supplementary file4 (PDF 6 KB)

Supplementary file5 (DOCX 31 KB)

Supplementary file6 (PDF 21 KB)

Supplementary file7 (PDF 21 KB)

## Declarations

### Data Availability

All data and material reported in this systematic review and meta-analysis are from peer-reviewed publications.

## References

[1] Moore DA, Jones B, Weakley J, Whitehead S, Till K (2022). The field and resistance training loads of academy rugby league players during a pre-season: comparisons across playing positions. PLoS One.

[2] Mcleod JC, Stokes T, Phillips SM (2019). Resistance exercise training as a primary countermeasure to age-related chronic disease. Front Physiol.

[3] Till K, Darrall-Jones J, Weakley JJ, Roe GA, Jones BL (2017). The influence of training age on the annual development of physical qualities within academy rugby league players. J Strength Cond Res.

[4] Weakley J, Till K, Darrall-Jones J, Roe GA, Phibbs PJ, Read DB (2019). Strength and conditioning practices in adolescent rugby players: relationship with changes in physical qualities. J Strength Cond Res.

[5] Morton RW, Oikawa SY, Wavell CG, Mazara N, Mcglory C, Quadrilatero J (2016). Neither load nor systemic hormones determine resistance training-mediated hypertrophy or strength gains in resistance-trained young men. J Appl Physiol.

[6] Banyard HG, Tufano JJ, Weakley JJS, Wu S, Jukic I, Nosaka K (2020). Superior changes in jump, sprint, and change-of-direction performance but not maximal strength following 6 weeks of velocity-based training compared with 1-repetition-maximum percentage-based training. Int J Sports Physiol Perform.

[7] Bird SP, Tarpenning KM, Marino FE (2005). Designing resistance training programmes to enhance muscular fitness. Sports Med.

[8] García-Ramos A, Ulloa-Díaz D, Barboza-González P, Rodríguez-Perea Á, Martínez-García D, Quidel-Catrilelbún M (2019). Assessment of the load-velocity profile in the free-weight prone bench pull exercise through different velocity variables and regression models. PLoS One.

[9] Pearson M, García-Ramos A, Morrison M, Ramirez-Lopez C, Dalton-Barron N, Weakley J (2020). Velocity loss thresholds reliably control kinetic and kinematic outputs during free weight resistance training. Int J Environ Res.

[10] Schoenfeld BJ, Peterson MD, Ogborn D, Contreras B, Sonmez GT (2015). Effects of low-vs. high-load resistance training on muscle strength and hypertrophy in well-trained men. J Strength Cond Res.

[11] Salmoni AW, Schmidt RA, Walter CB (1984). Knowledge of results and motor learning: a review and critical reappraisal. Psychol Bull.

[12] Wälchli M, Ruffieux J, Bourquin Y, Keller M, Taube W (2016). Maximizing performance: augmented feedback, focus of attention, and/or reward?. Med Sci Sports Exerc.

[13] Argus CK, Gill ND, Keogh JW, Hopkins WG (2011). Acute effects of verbal feedback on upper-body performance in elite athletes. J Strength Cond Res.

[14] Weakley J, Wilson K, Till K, Banyard H, Dyson J, Phibbs P (2020). Show me, tell me, encourage me: the effect of different forms of feedback on resistance training performance. J Strength Cond Res.

[15] Ok DP, Bae JY (2019). Accelerometer-based instantaneous feedback technology is as effective as coach’s supervision on the quantity and quality of resistance training sessions for university wrestling athletes. J Men Health.

[16] Weakley J, Wilson K, Till K, Darrall-Jones J, Roe G, Phibbs P (2017). Visual feedback maintains mean concentric barbell velocity, and improves motivation, competitiveness, and perceived workload in male adolescent athletes. J Strength Cond Res..

[17] Pérez-Castilla A, Jiménez-Alonso A, Cepero M, Miras-Moreno S, Rojas FJ, García-Ramos A (2020). Velocity performance feedback during ballistic training: which is the optimal frequency of feedback administration?. Mot Control.

[18] Jiménez-Alonso A, García-Ramos A, Cepero M, Miras-Moreno S, Rojas FJ, Pérez-Castilla A (2022). Effect of augmented feedback on velocity performance during strength-oriented and power-oriented resistance training sessions. J Strength Cond Res.

[19] Nagata A, Doma K, Yamashita D, Hasegawa H, Mori S (2020). The effect of augmented feedback type and frequency on velocity-based training-induced adaptation and retention. J Strength Cond Res.

[20] Randell AD, Cronin JB, Keogh JW, Gill ND, Pedersen MC (2011). Effect of instantaneous performance feedback during 6 weeks of velocity-based resistance training on sport-specific performance tests. J Strength Cond Res.

[21] Vanderka M, Bezák A, Longová K, Krcmár M, Walker S (2020). Use of visual feedback during jump-squat training aids improvement in sport-specific tests in athletes. J Strength Cond Res.

[22] Weakley J, Till K, Sampson J, Banyard H, Leduc C, Wilson K (2019). The effects of augmented feedback on sprint, jump, and strength adaptations in rugby union players following a four week training programme. Int J Sports Physiol Perform.

[23] Winchester JB, Porter JM, Mcbride JM (2009). Changes in bar path kinematics and kinetics through use of summary feedback in power snatch training. J Strength Cond Res.

[24] Page M, Mckenzie J, Bossuyt P, Boutron I, Hoffmann T, Mulrow C (2021). The prisma 2020 statement: an updated guideline for reporting systematic reviews. Syst Rev.

[25] Cooper H, Hedges LV, Valentine JC (2019). The handbook of research synthesis and meta-analysis.

[26] Steele J, Plotkin D, Van Every D, Rosa A, Zambrano H, Mendelovits B (2021). Slow and steady, or hard and fast? A systematic review and meta-analysis of studies comparing body composition changes between interval training and moderate intensity continuous training. Sports (Basel).

[27] Downs SH, Black N (1998). The feasibility of creating a checklist for the assessment of the methodological quality both of randomised and non-randomised studies of health care interventions. J Epidemiol Community Health.

[28] Crang ZL, Duthie G, Cole MH, Weakley J, Hewitt A, Johnston RD (2021). The validity and reliability of wearable microtechnology for intermittent team sports: a systematic review. Sports Med.

[29] Weakley J, Morrison M, García-Ramos A, Johnston R, James L, Cole M (2021). The validity and reliability of commercially available resistance training monitoring devices—a systematic review. Sports Med.

[30] Weakley J, Halson SL, Mujika I (2022). Overtraining syndrome symptoms and diagnosis in athletes: where is the research? A systematic review. Int J Sports Physiol Perform.

[31] Team R. Rstudio: integrated development for r, in Boston, MA (2022).

[32] Lüdecke D, Lüdecke MD, Calculator'from David BW. Package ‘esc’*.* R Package Version 0.5;12019 (2019).

[33] Viechtbauer W (2010). Conducting meta-analyses in r with the metafor package. J Stat Softw.

[34] Cohen J (2013). Statistical power analysis for the behavioral sciences.

[35] Weakley JJS, Till K, Read DB, Leduc C, Roe GB, Phibbs PJ (2021). Jump training in rugby union players: barbell or hexagonal bar?. J Strength Cond Res.

[36] Weakley J, Munteanu G, Cowley N, Johnston R, Morrison M, Gardiner C (2022). The criterion validity and between-day reliability of the perch for measuring barbell velocity during commonly used resistance training exercises. J Strength Cond Res.

[37] Weakley J, Chalkley D, Johnston R, García-Ramos A, Townshend A, Dorrell H (2020). Criterion validity, and interunit and between-day reliability of the flex for measuring barbell velocity during commonly used resistance training exercises. J Strength Cond Res.

[38] Janicijevic D, García-Ramos A, Lamas-Cepero JL, García-Pinillos F, Marcos-Blanco A, Rojas FJ, et al. Comparison of the two most commonly used gold-standard velocity monitoring devices (GymAware and T-Force) to assess lifting velocity during the free-weight barbell back squat exercise*.* Proc Inst Mech Eng P J Sport Eng Technol. 2021:17543371211029614.

[39] Gucciardi DF, Lines RL, Ntoumanis N (2022). Handling effect size dependency in meta-analysis. Int Rev Sport Exerc Psychol.

[40] López-López JA, Page MJ, Lipsey MW, Higgins JPT (2018). Dealing with effect size multiplicity in systematic reviews and meta-analyses. Res Synth Methods.

[41] Inthout J, Ioannidis JPA, Borm GF (2014). The Hartung–Knapp–Sidik–Jonkman method for random effects meta-analysis is straightforward and considerably outperforms the standard Dersimonian–Laird method. BMC Med Res Method.

[42] Hartung J, Knapp G (2001). On tests of the overall treatment effect in meta-analysis with normally distributed responses. Stat Med.

[43] Nakagawa S, Lagisz M, Jennions MD, Koricheva J, Noble DWA, Parker TH (2022). Methods for testing publication bias in ecological and evolutionary meta-analyses. Method Ecol Evol.

[44] Higgins J, Thomas J, Chandler J, Cumpston M, Li T, Page MJ (2022). Cochrane handbook for systematic reviews of interventions.

[45] Wilson KM, Helton WS, De Joux NR, Head JR, Weakley JJ (2017). Real-time quantitative performance feedback during strength exercise improves motivation, competitiveness, mood, and performance. Proc Hum Factors Ergon Soc.

[46] Jiménez-Alonso A, García-Ramos A, Cepero M, Miras-Moreno S, Rojas FJ, Pérez-Castilla A (2022). Velocity performance feedback during the free-weight bench press testing procedure: an effective strategy to increase the reliability and one repetition maximum accuracy prediction. J Strength Cond Res.

[47] Chalker WJ, Shield AJ, Opar DA, Rathbone EN, Keogh JW (2018). Effect of acute augmented feedback on between limb asymmetries and eccentric knee flexor strength during the nordic hamstring exercise. PeerJ.

[48] Winchester JB, Erickson TM, Blaak JB, Mcbride JM (2005). Changes in bar-path kinematics and kinetics after power-clean training. J Strength Cond Res.

[49] Sakadjian A, Panchuk D, Pearce AJ (2014). Kinematic and kinetic improvements associated with action observation facilitated learning of the power clean in Australian footballers. J Strength Cond Res.

[50] Ekblom M, Eriksson M (2012). Concurrent EMG feedback acutely improves strength and muscle activation. Eur J App Physiol.

[51] Hopper DM, Berg MA, Andersen H, Madan R (2003). The influence of visual feedback on power during leg press on elite women field hockey players. Phys Ther Sport.

[52] Campenella B, Mattacola CG, Kimura IF (2000). Effect of visual feedback and verbal encouragement on concentric quadriceps and hamstrings peak torque of males and females. Isokinet Exerc Sci.

[53] Kimura IF, Gulick DT, Lukasiewicz Iii WC (1999). Effect of visual feedback and verbal encouragement on eccentric quadriceps and hamstrings peak torque. Res Sport Med.

[54] Keller M, Lauber B, Gehring D, Leukel C, Taube W (2014). Jump performance and augmented feedback: immediate benefits and long-term training effects. Hum Mov Sci.

[55] Wilson KM, De Joux NR, Head JR, Helton WS, Dang JS, Weakley JJ (2018). Presenting objective visual performance feedback over multiple sets of resistance exercise improves motivation, competitiveness, and performance. Proc Hum Factors Ergon Soc.

[56] Weakley J, Ramirez-Lopez C, Mclaren S, Dalton-Barron N, Weaving D, Jones B (2019). The effects of 10%, 20%, and 30% velocity loss thresholds on kinetic, kinematic, and repetition characteristics during the barbell back squat. Int J Sports Physiol Perform.

[57] Garcia-Ramos A, Barboza-Gonzalez P, Ulloa-Diaz D, Rodriguez-Perea A, Martinez-Garcia D, Guede-Rojas F (2019). Reliability and validity of different methods of estimating the one-repetition maximum during the free-weight prone bench pull exercise. J Sports Sci.

[58] García-Ramos A, Janicijevic D, González-Hernández JM, Keogh JWL, Weakley J (2020). Reliability of the velocity achieved during the last repetition of sets to failure and its association with the velocity of the 1-repetition maximum. PeerJ.

[59] García-Ramos A, Jukic I, Weakley J, Janićijević D (2021). Bench press one-repetition maximum estimation through the individualised load-velocity relationship: comparison of different regression models and minimal velocity thresholds. Int J Sports Physiol Perf.

[60] González-Badillo JJ, Rodríguez-Rosell D, Sánchez-Medina L, Gorostiaga EM, Pareja-Blanco F (2014). Maximal intended velocity training induces greater gains in bench press performance than deliberately slower half-velocity training. Eur J Sport Sci.

[61] Behm DG, Sale DG (1993). Intended rather than actual movement velocity determines velocity-specific training response. J App Physiol.

[62] Cormie P, Mcguigan MR, Newton RU (2010). Adaptations in athletic performance after ballistic power versus strength training. Med Sci Sports Exerc.

[63] Morin J-B, Slawinski J, Dorel S, De Villareal ES, Couturier A, Samozino P (2015). Acceleration capability in elite sprinters and ground impulse: push more, brake less?. J Biomech.

[64] Nagahara R, Mizutani M, Matsuo A, Kanehisa H, Fukunaga T (2018). Association of sprint performance with ground reaction forces during acceleration and maximal speed phases in a single sprint. J Appl Biomech.

[65] Weakley J, Wilson K, Till K, Read D, Scantlebury S, Sawczuk T (2018). Visual kinematic feedback enhances velocity, power, motivation and competitiveness in adolescent female athletes. J Aust Strength Cond.

[66] Keller M, Lauber B, Gottschalk M, Taube W (2015). Enhanced jump performance when providing augmented feedback compared to an external or internal focus of attention. J Sports Sci.

[67] Weakley J, Mann B, Banyard H, Mclaren S, Scott T, Garcia-Ramos A (2021). Velocity-based training: from theory to application. Strength Cond J.

[68] Morrison M, Martin DT, Talpey S, Scanlan AT, Delaney J, Halson SL (2022). A systematic review on fitness testing in adult male basketball players: tests adopted, characteristics reported and recommendations for practice. Sports Med.

[69] Owen C, Till K, Phibbs P, Read DJ, Weakley J, Atkinson M (2023). A multidimensional approach to identifying the physical qualities of male English regional academy rugby union players; considerations of position, chronological age, relative age and maturation. Eur J Sport Sci.

[70] Weakley J, Black G, McLaren S, Scantlebury S, Suchomel T, McMahon E, Watts D, Read DB (2023). Testing and profiling athletes: recommendations for test selection, implementation, and maximizing information. Strength Cond J.

