# Supplementary material for: The Effect of Feedback on Resistance Training Performance and Adaptations: A Systematic Review and Meta-analysis
Source: Sports Med. 2023 Jul 6;53(9):1789–803. doi: 10.1007/s40279-023-01877-2 (PMC10432365; doi:10.1007/s40279-023-01877-2)
Supplement: Supplementary file 1 — Supplementary file1 (DOCX 13 KB) [file 40279_2023_1877_MOESM1_ESM.docx]

Supplementary File 1.

Search string

Scopus

( TITLE-ABS-KEY ( feedback OR "augmented feedback" ) AND TITLE-ABS-KEY ( "resistance training" OR "weight training" OR "strength training" OR "velocity-based" OR "resistance exercise" OR "Strength and conditioning" ) AND TITLE-ABS-KEY ( visual OR verbal OR encouragement OR "Performance feedback" OR "Augmented feedback" ) AND TITLE-ABS-KEY ( velocity OR power OR strength OR jump OR speed OR performance ) )

Pubmed/Medline

(feedback OR "augmented feedback" ) AND ( "resistance training" OR "weight training" OR "strength training" OR "velocity-based" OR "resistance exercise" OR "Strength and conditioning" ) AND ( visual OR verbal OR encouragement OR "Performance feedback" OR "Augmented feedback" ) AND ( velocity OR power OR strength OR jump OR speed OR performance )

CINAHL/SportDiscuss

( feedback OR "augmented feedback" ) AND ( "resistance training" OR "weight training" OR "strength training" OR "velocity-based" OR "resistance exercise" OR "Strength and conditioning" ) AND ( visual OR verbal OR encouragement OR "Performance feedback" OR "Augmented feedback" ) AND ( velocity OR power OR strength OR jump OR speed OR performance )
