# Supplementary material for: The Effect of Feedback on Resistance Training Performance and Adaptations: A Systematic Review and Meta-analysis
Source: Sports Med. 2023 Jul 6;53(9):1789–803. doi: 10.1007/s40279-023-01877-2 (PMC10432365; doi:10.1007/s40279-023-01877-2)
Supplement: Supplementary file 2 — Supplementary file2 (DOCX 24 KB) [file 40279_2023_1877_MOESM2_ESM.docx]

| **Electronic Supplementary File 2.** Modified Downs and Black scoring system. | | |
| --- | --- | --- |
| No. | Item | Score |
| 1 | Is the hypothesis/aim/objective of the study clearly stated? | 0-1 |
| 2 | Are the outcome measures clearly stated? | 0-1 |
| 3 | Are details of the participant characteristics clearly stated? | 0-1 |
| 4 | Are the interventions of interest clearly described? | 0-1 |
| 6 | Are the findings of the study clearly described? | 0-1 |
| 7 | Does the study provide estimates of the random variability in the data for the main outcomes? | 0-1 |
| 10 | Have actual statistical values been reported? | 0-1 |
| 16 | If any of the results were based on data dredging, was this made clear? | 0-1 |
| 18 | Were the statistical tests used appropriate? | 0-1 |
| 19 | Was compliance with the intervention reliable? | 0-1 |
| 20 | Were the outcome measures used valid and reliable? | 0-1 |
| 21 | Were subjects in different intervention groups recruited from the same population? | 0-1 |
| 22 | Were study subjects recruited over the same period of time? | 0-1 |
| 23 | Were study subjects randomised to intervention groups? | 0-1 |
| 24 | Was the randomised intervention assignment concealed from both patients and health care staff until recruitment was complete and irrevocable? | 0-1 |
| 26 | Were losses of patients to follow-up taken into account? | 0-1 |
| 27 | Did the study have sufficient power to detect a clinically important effect where the probability value for a difference being due to chance is less than 5%? | 0-1 |
