# Supplementary material for: The Effect of Feedback on Resistance Training Performance and Adaptations: A Systematic Review and Meta-analysis
Source: Sports Med. 2023 Jul 6;53(9):1789–803. doi: 10.1007/s40279-023-01877-2 (PMC10432365; doi:10.1007/s40279-023-01877-2)

Study	Duration	Test	Unit	Control Group					Feedback Group					Estimate [95% CI]
					Pre		Post			Pre		Post		
				n	*M*	*SD*	*M*	*SD*	n	*M*	*SD*	*M*	*SD*	
Nagata et al. 2020 [19]	4	30kg Squat Jump	m/s	8	1.36	0.04	1.30	0.06	9	1.40	0.05	1.52	0.06	3.14 [1.68, 4.60]
Weakley et al. 2019 [22]	4	Broad Jump	cm	12	231.00	23.00	242.00	22.00	16	227.00	23.00	240.00	26.00	0.08 [−0.67, 0.83]
Weakley et al. 2019 [22]	4	Countermovement Jump	cm	12	41.50	5.10	42.00	8.30	16	40.20	8.10	41.60	8.40	0.11 [−0.64, 0.86]
Randell et al. 2011 [20]	6	Broad Jump	cm	6	258.00	20.00	259.00	20.00	7	250.00	16.00	256.00	15.00	0.26 [−0.83, 1.36]
Vanderka et al. 2020 [21]	6	Countermovement Jump	cm	12	44.40	6.30	42.30	6.30	13	49.30	4.60	52.20	5.10	0.86 [0.04, 1.69]
Vanderka et al. 2020 [21]	6	Squat Jump	cm	12	40.10	5.90	39.00	5.30	13	44.10	4.20	46.80	4.90	0.72 [−0.09, 1.53]
Randell et al. 2011 [20]	6	Vertical Jump	cm	6	66.00	6.00	67.00	1.00	7	61.00	6.00	64.00	7.00	0.30 [−0.79, 1.40]
Model for All Outcomes														0.94 [−0.55, 2.44]

Heterogeneity: I^2 (effect) = 0%, I^2 (study) = 85.8%, I^2 (total) = 85.8%

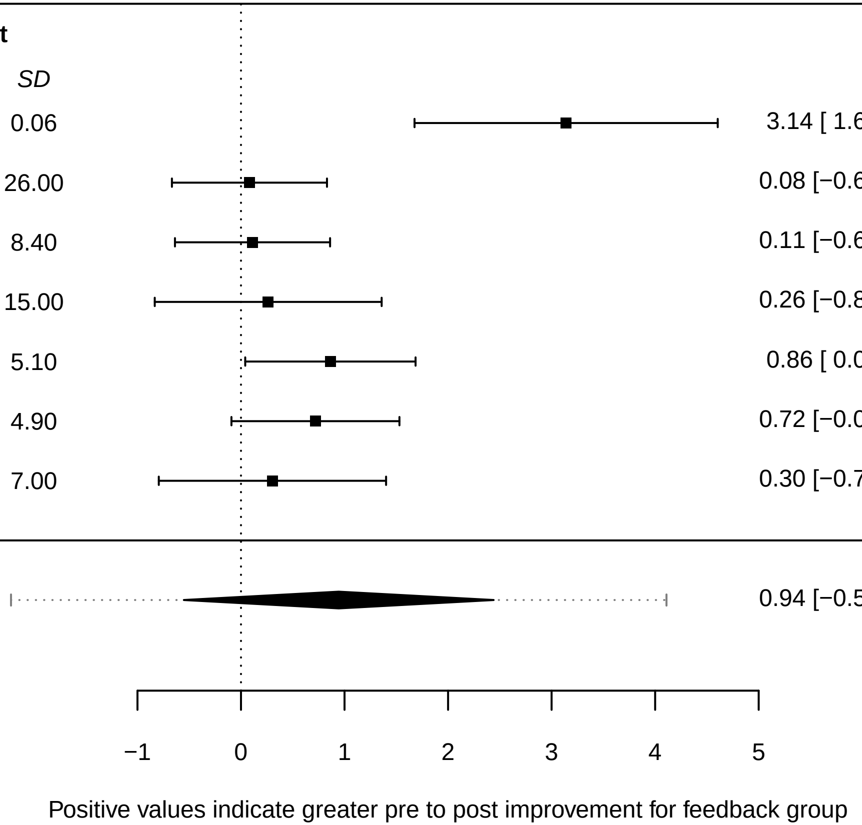

Supplement: Supplementary file 3 — Supplementary file3 (PDF 6 KB) [file 40279_2023_1877_MOESM3_ESM.pdf]
