# Supplementary figures and images for: The Effect of Feedback on Resistance Training Performance and Adaptations: A Systematic Review and Meta-analysis
Source: Sports Med. 2023 Jul 6;53(9):1789–803. doi: 10.1007/s40279-023-01877-2 (PMC10432365; doi:10.1007/s40279-023-01877-2)

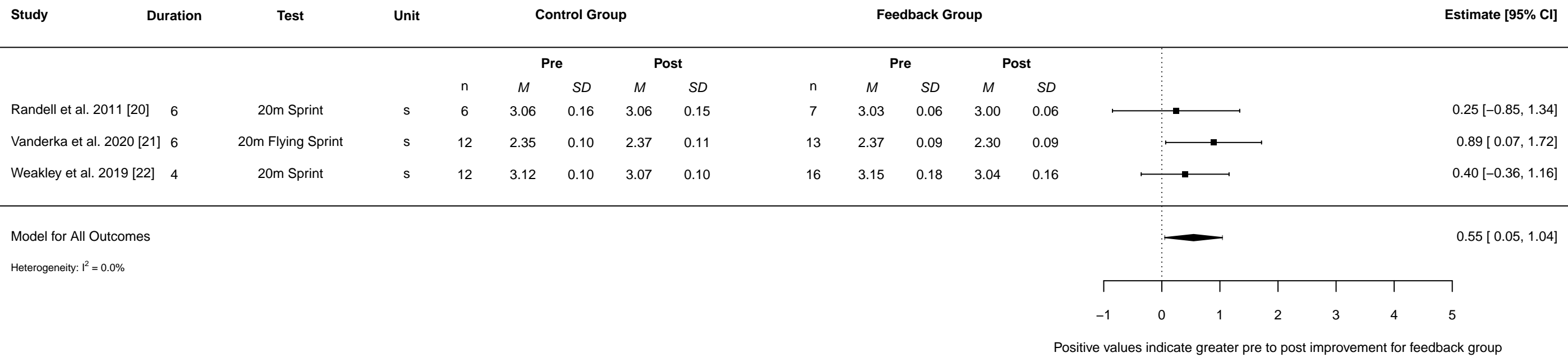

Supplement: Supplementary file 4 — Supplementary file4 (PDF 6 KB) [file 40279_2023_1877_MOESM4_ESM.pdf]

Electronic Supplementary Material File S6  
Funnel plot including all studies

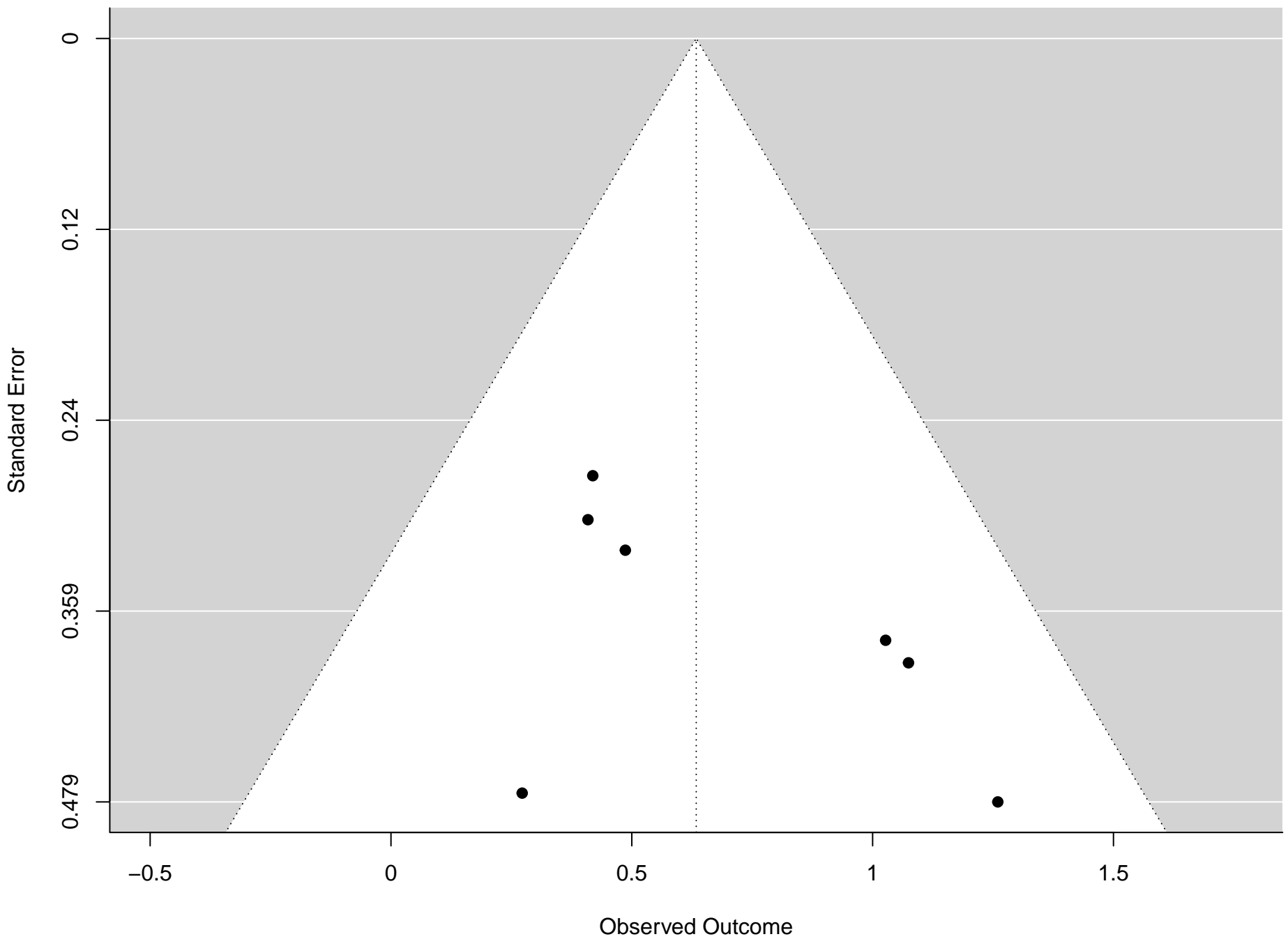

Supplement: Supplementary file 6 — Supplementary file6 (PDF 21 KB) [file 40279_2023_1877_MOESM6_ESM.pdf]

Electronic Supplementary Material File S7  
Funnel plot with trim and fill

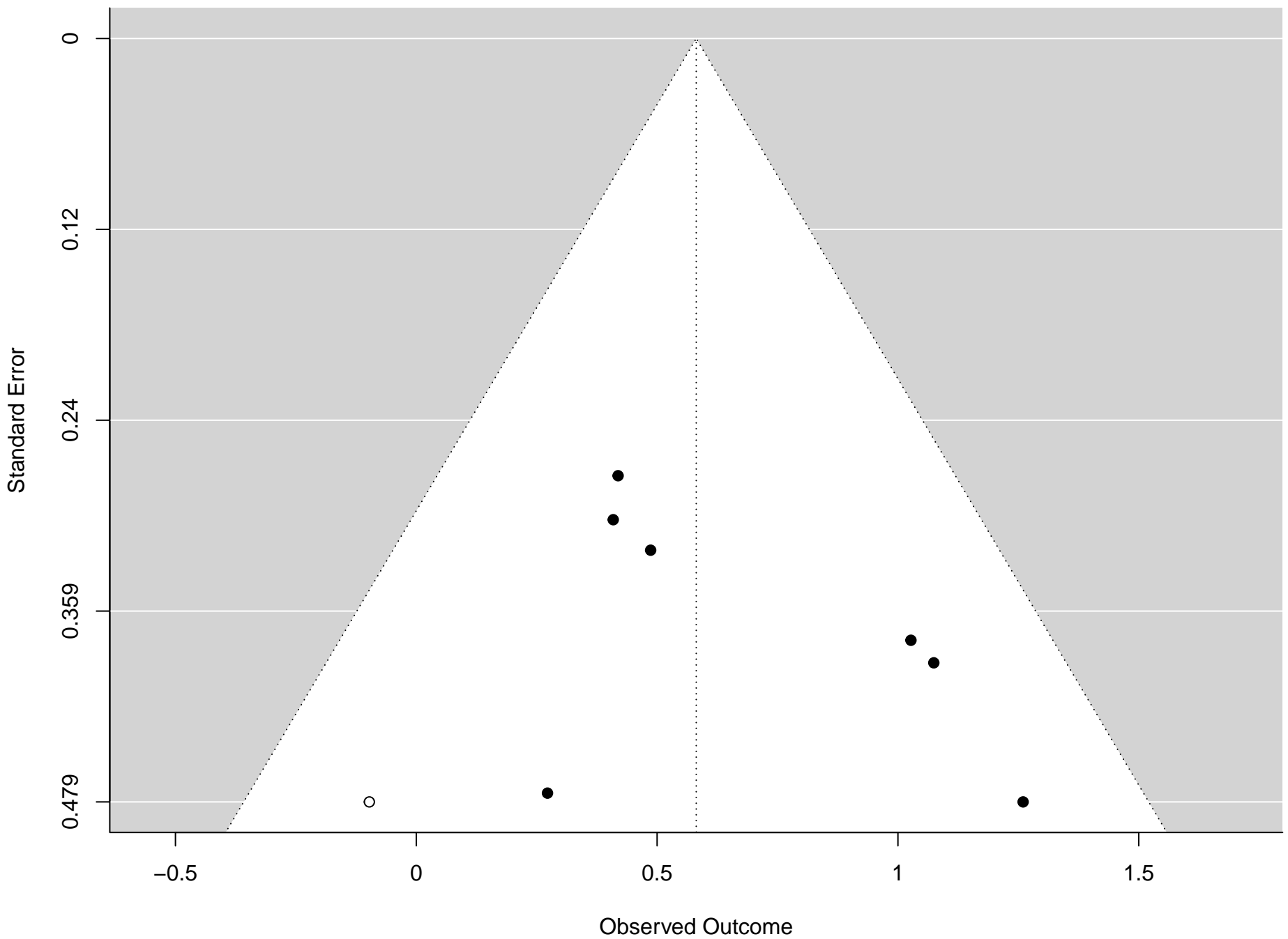

Supplement: Supplementary file 7 — Supplementary file7 (PDF 21 KB) [file 40279_2023_1877_MOESM7_ESM.pdf]
