# Supplementary material for: The Effect of Feedback on Resistance Training Performance and Adaptations: A Systematic Review and Meta-analysis
Source: Sports Med. 2023 Jul 6;53(9):1789–803. doi: 10.1007/s40279-023-01877-2 (PMC10432365; doi:10.1007/s40279-023-01877-2)
Supplement: Supplementary file 5 — Supplementary file5 (DOCX 31 KB) [file 40279_2023_1877_MOESM5_ESM.docx]

**Electronic Supplementary Material 5.** Modified Downs and Black reporting outcomes.

|  | Item Number | | | | | | | | | | | | | | | | |  |
| --- | --- | --- | --- | --- | --- | --- | --- | --- | --- | --- | --- | --- | --- | --- | --- | --- | --- | --- |
| **Study Title** | 1 | 2 | 3 | 4 | 6 | 7 | 10 | 16 | 18 | 19 | 20 | 21 | 22 | 23 | 24 | 26 | 27 | **Total** |
| **Acute studies** |  |  |  |  |  |  |  |  |  |  |  |  |  |  |  |  |  |  |
| Argus et al., 2011 [13] | 1 | 1 | 1 | 1 | 1 | 1 | 1 | 1 | 1 | 1 | 1 | 1 | 1 | 1 | 1 | 1 | 0 | **16** |
| Campenella et al., 2000 [52] | 1 | 1 | 1 | 1 | 1 | 1 | 0 | 1 | 1 | 1 | 1 | 1 | 1 | 1 | 1 | 1 | 0 | **15** |
| Chalker et al., 2018 [47] | 1 | 1 | 1 | 1 | 1 | 0 | 0 | 1 | 1 | 1 | 1 | 0 | 1 | 0 | 0 | 1 | 0 | **11** |
| Ekblom and Eriksson 2012 [50] | 1 | 0 | 1 | 1 | 1 | 0 | 0 | 1 | 1 | 1 | 1 | 1 | 1 | 1 | 1 | 1 | 0 | **13** |
| Hopper et al., 2003 [51] | 1 | 0 | 1 | 1 | 0 | 1 | 1 | 1 | 0 | 1 | 1 | 1 | 1 | 1 | 1 | 1 | 1 | **14** |
| Jiménez-Alonso et al. 2022a [18] | 1 | 1 | 1 | 1 | 1 | 1 | 1 | 1 | 1 | 1 | 1 | 1 | 1 | 1 | 1 | 1 | 0 | **16** |
| Jiménez-Alonso et al. 2022b [46] | 1 | 1 | 1 | 1 | 1 | 1 | 1 | 1 | 1 | 1 | 1 | 1 | 1 | 1 | 1 | 1 | 0 | **16** |
| Kimura et al., 1999 [53] | 1 | 1 | 0 | 1 | 1 | 1 | 1 | 1 | 1 | 1 | 1 | 1 | 1 | 1 | 1 | 1 | 0 | **15** |
| Ok & Bae 2019 [15] | 0 | 1 | 1 | 0 | 1 | 1 | 1 | 1 | 0 | 0 | 0 | 1 | 1 | 0 | 0 | 1 | 0 | **9** |
| Pérez-Castilla et al., 2019 [17] | 1 | 1 | 1 | 1 | 1 | 1 | 1 | 1 | 1 | 1 | 1 | 1 | 1 | 1 | 1 | 1 | 0 | **16** |
| Weakley et al., 2020 [14] | 1 | 1 | 1 | 1 | 1 | 1 | 1 | 1 | 1 | 1 | 1 | 1 | 1 | 1 | 1 | 1 | 0 | **16** |
| Weakley et al., 2019a [16] | 1 | 1 | 1 | 1 | 1 | 1 | 1 | 1 | 1 | 1 | 1 | 1 | 1 | 1 | 1 | 1 | 0 | **16** |
| Wilson et al., 2018 [45] | 1 | 1 | 1 | 1 | 1 | 1 | 1 | 1 | 1 | 1 | 1 | 1 | 1 | 1 | 1 | 1 | 0 | **16** |
|  |  |  |  |  |  |  |  |  |  |  |  |  |  |  |  |  |  |  |
| **Chronic Studies** |  |  |  |  |  |  |  |  |  |  |  |  |  |  |  |  |  |  |
| Nagata et al., 2020 [19] | 1 | 1 | 1 | 1 | 1 | 1 | 1 | 1 | 1 | 1 | 0 | 1 | 1 | 0 | 1 | 1 | 0 | **14** |
| Randell et al., 2011 [20] | 1 | 1 | 1 | 1 | 1 | 1 | 1 | 1 | 1 | 0 | 1 | 1 | 1 | 1 | 1 | 0 | 0 | **14** |
| Sakadjian et al., 2014 [49] | 0 | 0 | 0 | 0 | 1 | 0 | 1 | 1 | 1 | 1 | 0 | 1 | 1 | 0 | 1 | 1 | 0 | **9** |
| Vanderka et al., 2020 [21] | 1 | 1 | 1 | 1 | 1 | 1 | 1 | 1 | 1 | 0 | 1 | 1 | 1 | 1 | 1 | 0 | 0 | **14** |
| Weakley et al., 2019b [22] | 1 | 1 | 1 | 1 | 1 | 1 | 1 | 1 | 1 | 1 | 1 | 1 | 1 | 1 | 1 | 1 | 0 | **16** |
| Winchester et al., 2005 [48] | 0 | 0 | 0 | 0 | 1 | 1 | 0 | 1 | 0 | 1 | 1 | 1 | 1 | 0 | 0 | 0 | 0 | **7** |
| Winchester et al., 2009 [23] | 0 | 1 | 0 | 0 | 1 | 1 | 0 | 1 | 1 | 1 | 1 | 1 | 1 | 1 | 1 | 0 | 0 | **11** |
